# Central nervous system concentrations of nano- and microplastics and risk of amyotrophic lateral sclerosis

**DOI:** 10.1093/braincomms/fcag296

**Published:** 2026-08-03

**Authors:** Marco Vinceti, Margherita Ferrante, Tommaso Filippini, Lauren A Wise, Teresa Urbano, Roberta Bedin, Giulia Gianferrari, Paola Rapisarda, Eloise Pulvirenti, Gea Oliveri Conti, Jessica Mandrioli

**Affiliations:** Environmental, Genetic and Nutritional Epidemiology Research Center (CREAGEN), Department of Biomedical, Metabolic and Neural Sciences, University of Modena and Reggio Emilia, Modena 41125, Italy; Department of Epidemiology, Boston University School of Public Health, Boston, MA 02118, USA; Section of Public Health, Department of Medical Sciences, Surgical and Advanced Technologies ‘G.F. Ingrassia’, University of Catania, Catania 95123, Italy; Environmental, Genetic and Nutritional Epidemiology Research Center (CREAGEN), Department of Biomedical, Metabolic and Neural Sciences, University of Modena and Reggio Emilia, Modena 41125, Italy; School of Public Health, University of California Berkeley, Berkeley, CA 94704, USA; Department of Epidemiology, Boston University School of Public Health, Boston, MA 02118, USA; Environmental, Genetic and Nutritional Epidemiology Research Center (CREAGEN), Department of Biomedical, Metabolic and Neural Sciences, University of Modena and Reggio Emilia, Modena 41125, Italy; Environmental, Genetic and Nutritional Epidemiology Research Center (CREAGEN), Department of Biomedical, Metabolic and Neural Sciences, University of Modena and Reggio Emilia, Modena 41125, Italy; Environmental, Genetic and Nutritional Epidemiology Research Center (CREAGEN), Department of Biomedical, Metabolic and Neural Sciences, University of Modena and Reggio Emilia, Modena 41125, Italy; Department of Neurosciences, Modena Policlinico University Hospital, Modena 41126, Italy; Section of Public Health, Department of Medical Sciences, Surgical and Advanced Technologies ‘G.F. Ingrassia’, University of Catania, Catania 95123, Italy; Section of Public Health, Department of Medical Sciences, Surgical and Advanced Technologies ‘G.F. Ingrassia’, University of Catania, Catania 95123, Italy; Section of Public Health, Department of Medical Sciences, Surgical and Advanced Technologies ‘G.F. Ingrassia’, University of Catania, Catania 95123, Italy; Environmental, Genetic and Nutritional Epidemiology Research Center (CREAGEN), Department of Biomedical, Metabolic and Neural Sciences, University of Modena and Reggio Emilia, Modena 41125, Italy; Department of Neurosciences, Modena Policlinico University Hospital, Modena 41126, Italy

**Keywords:** amyotrophic lateral sclerosis, microplastics, nanoplastics

## Abstract

Growing evidence suggests that microplastics, particularly nanoplastics, may be neurotoxic. However, there are few studies of neurologic diseases, especially amyotrophic lateral sclerosis (ALS). In a hospital-based case-control study, we measured *in vivo* concentrations of nano- and microplastics (size 0.1–10 µm) in serum and CSF among 24 newly diagnosed cases of ALS and 20 controls. Adjusting for sex and age, we found a strong positive association between serum and CSF microplastic concentrations and higher microplastic concentrations in the CSF and serum of ALS cases compared with controls. In spline regression analyses, CSF microplastic concentrations showed a positive monotonic association with odds of ALS. Serum microplastic concentrations also showed a positive association with ALS, but only above a certain threshold. Among ALS cases, serum—but not CSF—microplastic concentrations were positively associated with neurofilament light chain, a biomarker of neuroaxonal damage. Given the case-control design, we cannot rule out reverse causation (i.e. that ALS-related factors caused greater bioaccumulation of microplastics) or the possibility that the association reflects changes in lifestyle or other chemical exposures. However, the observed pattern raises the possibility of an aetiologic role of nano- and microplastics in motor neuron degeneration and indicates higher CNS concentrations of these chemicals in ALS cases.

## Graphical Abstract

**Graphical Abstract:**
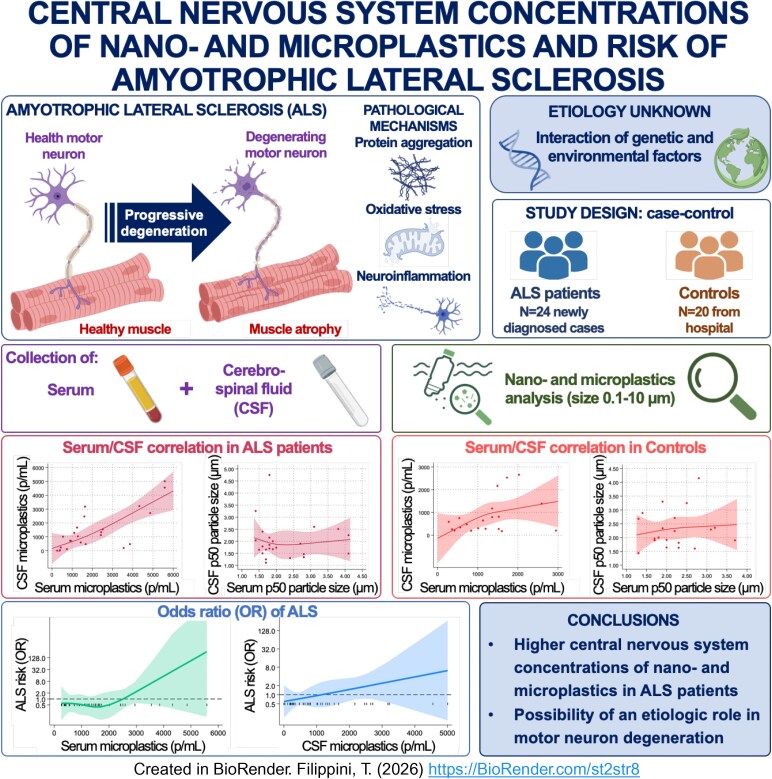

## Teaser

Vinceti *et al*. report higher microplastic (sized 0.1–10 µm) concentrations in serum and CSF of patients with amyotrophic lateral sclerosis compared with controls, with serum levels non-linearly associated with disease risk. Though residual confounding cannot be excluded, the possible role of small microplastics in this neurodegenerative disease warrants further investigation.

## Introduction

There is growing concern about the presence of microplastics, i.e. plastics with diameters < 5 mm,^1,2^ in the human body, and their effects on human health. Interest is even greater for the smallest microplastics, nanoplastics, with diameters < 1000 nm.^1-4^ Studies have detected concentrations of microplastics in the autoptic brain^5,6^ and CSF.^7-11^ Additional studies have shown potential for microplastics and nanoplastics to exert toxic effects on the human brain, and more generally on the CNS, thereby bringing attention to their possible role in neurodegenerative disease.^12-16^ Most data on the role of microplastics in neurodegenerative disease come from animal studies,^16-21^ and, to our knowledge, no study has investigated the relation between concentrations of microplastics in the CNS and the risk of amyotrophic lateral sclerosis (ALS), a severe CNS disease affecting motor neurons that remains of substantially unknown aetiology. Amyotrophic lateral sclerosis affects 2–12 individuals per 100 000 worldwide,^22^ has a higher incidence and prevalence in high-income countries, but is increasing at the highest rate in low- and middle-income countries.^23^

Given the availability of new analytical techniques for the measurement of microplastics of extremely small size in blood and CSF, we investigated the relation between CNS concentrations of nano- and microplastics in the 0.1–10 µm size range and the risk of amyotrophic lateral sclerosis from patients and healthy controls in a Northern Italian population.

## Materials and methods

### Study population

This study was conducted at the ALS Center of the Department of Neurology, Modena University Hospital in Northern Italy. Among patients newly diagnosed with amyotrophic lateral sclerosis between 1 January 2018 and 31 December 2022, we included 24 with definite or probable amyotrophic lateral sclerosis according to the revised El Escorial Criteria and with paired CSF and serum samples collected at the time of diagnostic lumbar puncture. In these patients, the median time from symptom onset to lumbar puncture was 12.5 months [interquartile range (IQR): 8.9–14.5 months]. We followed cases of amyotrophic lateral sclerosis for a mean of 48 months (range: 9–85), systematically collecting demographic and clinical data, including age at onset, sex, site of onset, body mass index (BMI), the Revised ALS Functional Rating Scale (ALSFRS-R) at diagnosis and its changes over time (disease progression rate)^24^ and a survival endpoint, i.e. death or tracheostomy, whichever occurred first. Genetic data on the four main ALS-associated genes (*C9orf72*, *SOD1*, *FUS* and *TARDBP*) were available for 20 of 24 cases.

The control group consisted of 20 individuals who underwent lumbar puncture and blood sampling for suspected neurological conditions that were subsequently ruled out after comprehensive diagnostic work-up. Based on neurological evaluation, neuroimaging and standard CSF analyses performed as part of the diagnostic work-up, controls had no clinical evidence of degenerative, inflammatory, infectious or systemic disorders known to affect CNS homeostasis or plausibly related to microplastic exposure, based on the literature. The most common diagnoses among controls were headache and, less frequently, symptomatic paraesthesias, visual disturbances, asthenia or psychomotor slowing, with negative diagnostic work-up for neurological disease. BMI was not routinely recorded at hospital admission and was retrieved only for six controls. In these subjects, we collected and processed CSF and serum samples using the same standardized procedures applied to cases.

In all participants, we collected venous blood samples using standard phlebotomy techniques and stored 1 mL of serum in serum separator tubes. For CSF, we performed lumbar punctures using standard clinical procedures, i.e. atraumatic needles of 25–22 gauge. We immediately anonymized both serum and CSF samples using an alphanumeric code upon their arrival at the laboratory, located at the same University Hospital, and processed within 30 min. We performed centrifugation at 2500× g for 10 min at controlled room temperature and aliquoted the sample supernatant into polypropylene sterile vials, which were kept frozen at −80°C at the Modena Neurobiobank. Each sample was then transported deep frozen on dry ice by air courier to the Laboratory of Catania University Hospital and transferred to glass vessels for microplastic analysis.

Overall, cases and controls came from the general population of the Modena and Reggio Emilia provinces.

All participants provided written informed consent to share their samples and clinical information for research purposes. The study was approved by the local Ethics Committee (No. 219/2024) and conducted in accordance with the Declaration of Helsinki.

### Laboratory determinations

In serum and CSF samples, we measured nano- and microplastics (hereafter referred to as ‘microplastics’) using an advanced extraction protocol we developed and patented at the University of Catania—European Patent EP No. 3788344/2022.^25-27^ Briefly, we transferred 1 mL from each serum and CSF sample into pre-cleaned borosilicate glass vessels and then subjected it to acid digestion, and we carried out digestion in an open-vessel graphite block system under controlled thermal conditions. After complete matrix degradation, we applied an organic liquid–liquid extraction, and we dispensed the concentrated extracts onto 25 mm aluminium scanning electron microscope (SEM) stubs and allowed to dry. Each stub was then coated with a thin conductive layer of pure gold using an automated sputter coater (Model 108 Auto Sputter Coater, Cressington Ltd., UK). We applied the same methodology to the serum samples.

We then performed a microscopic examination using a Hitachi S 4000 SEM equipped with an energy-dispersive X-ray (EDX) microanalysis system. This integrated SEM/EDX platform enabled high-resolution morphological assessment and elemental characterization. Quantification was carried out using MICROPLAST software (version 1.0), which provided particle counts (expressed as particles/mL) and size distribution parameters, with a limit of detection of 0.1 µm. In our study, we focused only on particles < 10 µm.

In addition to particle-based analyses, we assessed SEM images to identify the presence of synthetic microfibres, following the morphological criteria adopted by the World Health Organization.^28^ According to these frameworks, synthetic microfibres are defined by (i) length, typically > 5 µm (with some definitions extending up to several millimetres for fibrous microplastics); (ii) diameter, generally < 3 µm or otherwise markedly thin (often < 50  µm or < 10  µm depending on the regulatory context); and (iii) aspect ratio (L/D) > 3, indicating that the fibre must be at least three times longer than its diameter. No particles consistent with synthetic microfibres could be detected. In the analytical phase, we applied strict contamination-control procedures throughout the workflow. Laboratory personnel wore nitrile gloves and cotton laboratory coats, and all operations were conducted in a controlled-access clean room under a horizontal laminar flow hood. We processed reagent blanks in parallel with each analytical batch to verify the absence of background contamination.

We quantified neurofilament light chain (NfL), a biomarker of neuroaxonal damage released following axonal injury and measurable in both CSF and blood, which may also increase in acute neurological conditions.^29^ In amyotrophic lateral sclerosis, NfL is widely used as a biomarker of disease severity and progression, reflecting degeneration of motor neuron axons, particularly along the corticospinal tract.^30,31^ We measured NfL by using an automated next-generation ELISA platform (Ella™ Simple Plex assay technology, Bio-Techne ProteinSimple, San Jose, CA, USA), according to the manufacturer’s instructions and as previously described.^32,33^ Samples were loaded into the cartridges using 1:2 dilution for both serum and CSF NfL assay. We also determined neutrophil-to-lymphocyte ratio (NLR) at diagnosis of amyotrophic lateral sclerosis, a parameter associated with disease progression and survival.^34,35^

### Data analysis

We calculated the median and IQR of continuous variables. We assessed the correlation of microplastic concentrations between serum and CSF using a linear regression model and non-linear model based on restricted cubic splines with three knots at fixed percentiles (10th, 50th and 90th), adjusting for sex and age. In the latter analysis, we winsorized the highest value in serum to reduce the influence of the outliers.

We used logistic regression models to estimate odds ratios (ORs) and 95% confidence intervals (CIs), with and without adjustment for sex and age. In categorical models, we calculated risk of amyotrophic lateral sclerosis according to increasing tertile exposure (based on distribution among controls), using the lowest tertile as the reference. In non-linear models, we used restricted cubic splines using either three or four knots at fixed percentiles, calculated based on the distribution in control subjects and according Harrell’s recommendations (10th, 50th and 90th or 5th, 35th, 65th and 95th), using the Akaike information criterion to select the number of knots.

Among cases of amyotrophic lateral sclerosis, we used Cox proportional hazards regression to estimate hazard ratios (HRs) and 95% CIs for the association between microplastic concentrations and tracheostomy-free survival from symptom onset as well as ALSFRS-R score at diagnosis and disease progression rate.

### Data availability

The data that support the findings of this study are not publicly available since their containing information could compromise the privacy of research participants.

## Results

The median age was 55.8 years among the 24 cases of amyotrophic lateral sclerosis (7 females—29%) and 55.2 years among the 20 controls (12 females—60%). Among cases, the median diagnostic delay was 7.0 months (IQR: 5.9–8.5), disease onset was bulbar in four cases, the median ALSFRS-R score at diagnosis was 43 (IQR: 40–44) and the median disease progression rate was 0.495 points/month (IQR: 0.325–0.670). The median tracheostomy-free survival was 48.0 months (IQR: 27.9–58.5). Two patients carried known ALS-associated mutations: one harboured a mutation in the *SOD1* gene, while another carried a *C9orf72* repeat expansion.

In the overall study population, particle sizes ranged from 0.54 to 9.5 µm in serum (median value 1.66) and from 0.5 to 7.3 µm in CSF (median value 1.70), except for undetectable microplastic content in the CSF samples from two cases who had measurable levels in serum. The mean microplastic concentration was 1795.662 p/mL, while the median and IQR were 1359.7 and 734.83–2226.26, respectively.

**Figure 1:**
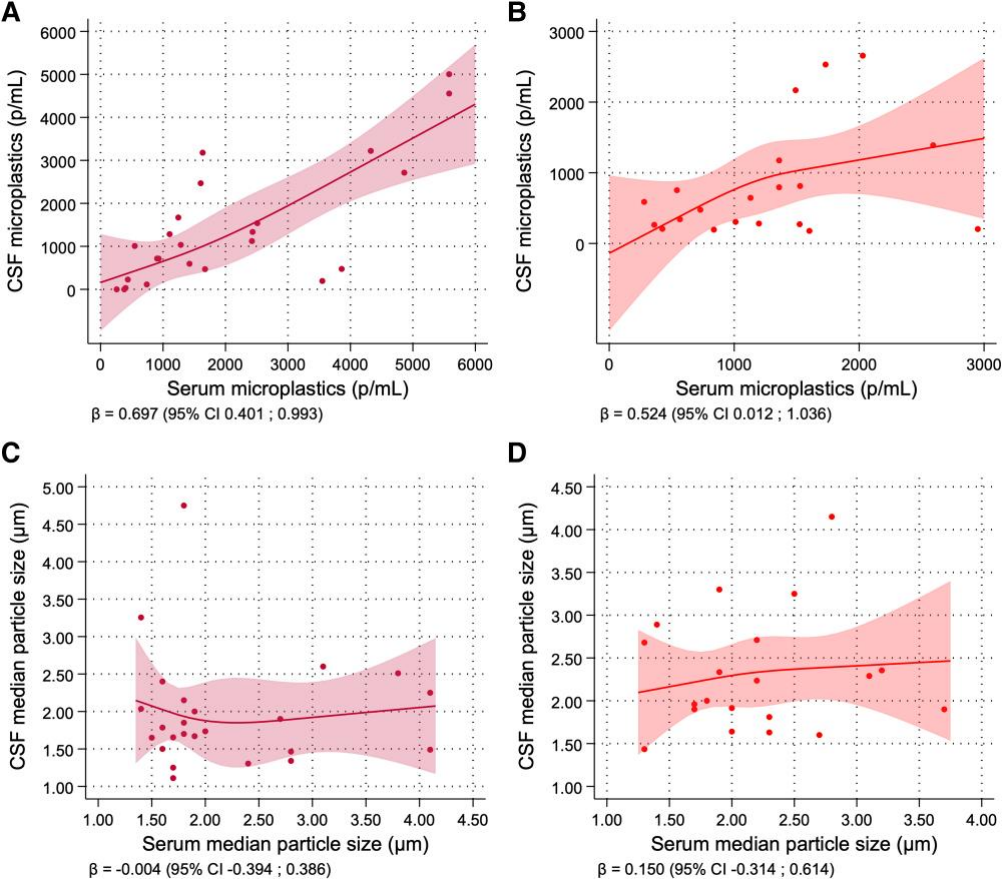
**Association of microplastic concentrations and size between serum and CSF.** Association between serum and CSF microplastic concentrations in ALS patients (**A**, *N* = 24) and controls (**B**, *N* = 20) and between serum and CSF particle size in ALS patients (**C**) and controls (**D**). The solid lines indicate the multivariable analysis; the shaded areas represent upper and lower CI limits. Dots represent individual observations.

CSF microplastic concentrations were strongly positively associated with serum concentrations in cases and controls, adjusting for age and sex, with a nearly linear pattern particularly among the former (Fig. 1). The linear regression coefficient of the association (β) was 0.667 (95% CI 0.466–0.868) in the overall study population after adjustment for sex and age, with little difference by case status (0.697–95% CI 0.401–0.993 in controls, 0.524–95% CI 0.012–1.036 in cases). With respect to the distribution of particle size in CSF relative to serum, we found a substantially flat, almost linear, association both in cases and controls (Fig. 1).

Older age was weakly associated with lower microplastic levels in both matrices. Among controls, females had higher microplastic concentration than males, while this did not occur in cases (Table 1). Among cases, serum microplastic concentrations were inversely correlated with BMI (β −0.094, 95% CI −0.200, 0.013 in serum, −0.089, 95% CI −0.204, 0.025 in CSF), the same pattern observed in controls though based on six valid observations only (β −0.397, 95% CI −0.890, 0.097 in serum, −0.608, 95% CI −1.761, 0.545 in CSF). The median BMI was substantially similar in cases (24.2) than controls (24.9). Microplastic concentrations in the four ALS patients with bulbar onset were higher in serum but lower in CSF compared with what was observed in patients with spinal-onset ALS.

**Table 1:** Microplastic concentrations in serum and CSF of study participants

Microplastics	ALS patients—median (IQR)		Controls—median (IQR)	
	Males (*n* = 17)	Females (*n* = 7)	Males (*n* = 8)	Females (*n* = 12)
Serum (p/mL)	1603.8 (931.0–3550.0)	1284.6 (436.4–2510.1)	1104.6 (631.3–1507.9)	1359.7 (647.4–1666.6)
CSF (p/mL)	1009.1 (469.8–2468.2)	1034.9 (227.5–1538.7)	293.6 (239.2–1377.9)	699.8 (302.9–993.8)

**Figure 2:**
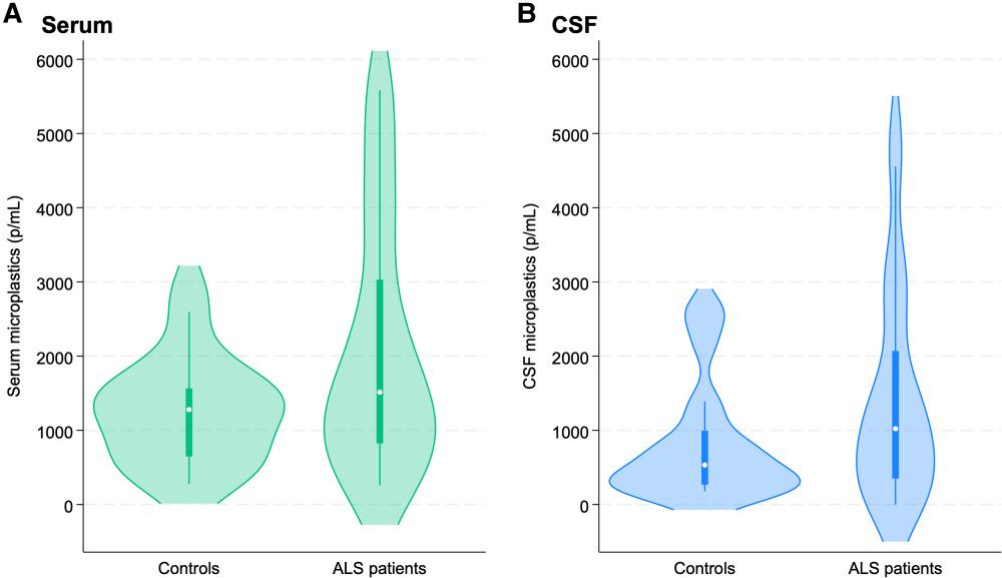
**Serum and CSF microplastic concentrations in ALS cases and controls.** Violin plots showing microplastic concentrations in serum (**A**) and CSF (**B**) of ALS patients (*N* = 24) and controls (*N* = 20).

Median microplastic levels in serum were higher among cases (1513.7 p/mL, IQR: 822.7–3030.0) than controls (1278.7 p/mL, IQR: 647.4–1564.7), while corresponding concentrations in CSF exhibited an even larger difference, with values of 1022.0 p/mL (IQR: 348.7–2069.5) among cases and 532.1 p/mL (IQR: 267.9–993.8) among controls (Fig. 2). In one of the two cases carrying a disease-related gene mutation (*SOD1*), microplastic concentrations in serum and CSF were considerably higher than their respective median values in all patients, i.e. 5381 and 5005 p/mL, respectively.

Patients’ CSF microplastic concentrations were higher among cases than controls in both sexes, while in serum, this was true only for males.

Using the distribution of microplastic concentrations in controls to define exposure categories, odds of amyotrophic lateral sclerosis were higher in the upper tertile of serum and CSF microplastic concentrations before and after adjustment for sex and age, with no increase in the middle tertile (Table 2). The association was more pronounced for CSF concentrations.

**Table 2:** OR with 95% CIs of ALS according to tertile of microplastic concentrations in controls

Exposure category	Cases/controls	Median (p/mL)	Crude OR (95% CI)	adj-OR (95% CI)^a^
Serum microplastics				
Tertile 1-Referent	6/6	430.8	1.0	1.0
Tertile 2	6/7	1197.8	0.9 (0.2–4.1)	0.7 (0.2–3.9)
Tertile 3	12/7	2433.9	1.7 (0.4–7.4)	1.7 (0.3–8.9)
CSF microplastics				
Tertile 1-Referent	6/6	194.6	1.0	1.0
Tertile 2	5/7	532.1	0.7 (0.1–3.6)	0.7 (0.1–3.7)
Tertile 3	13/7	1604.7	1.9 (0.4 -8.0)	2.2 (0.5–11.0)

^a^Adjusted for age and sex.

**Figure 3:**
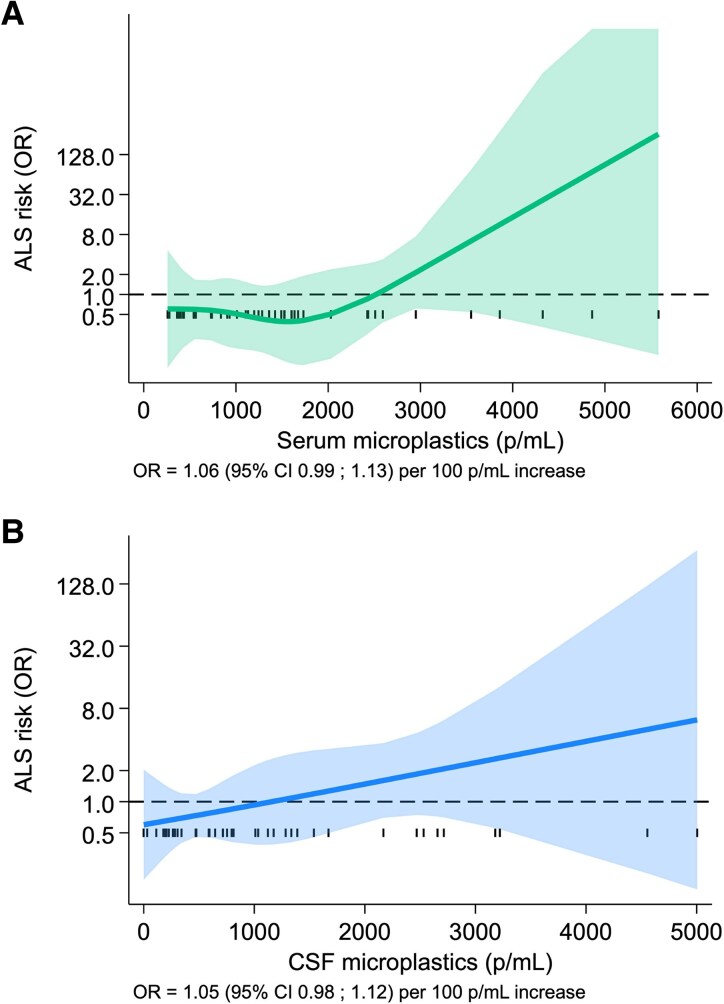
**ALS risk at increasing serum and CSF microplastic concentrations.** OR of ALS according to microplastic concentrations in serum (**A**) and in CSF (**B**). The solid lines indicate the OR from multivariable analysis; the shaded areas represent upper and lower CI limits. Vertical black lines represent individual observations. Analysis carried out on *N* = 44 subjects, *N* = 24 ALS patients and *N* = 20 controls.

When evaluating ORs for amyotrophic lateral sclerosis across the entire range of exposure in dose–response analysis, adjusted for sex and age, there was evidence of a positive and linear association with odds at concentrations above 2500 p/mL in serum (matrix in which we winsorized concentrations above 6000 p/mL), while there was no evidence of excess odds below this cut point (Fig. 3). At the lowest range of exposure, there was a slight indication of an inverse association between exposure and odds of amyotrophic lateral sclerosis. The relation between CSF microplastic concentrations and odds of amyotrophic lateral sclerosis was also positive and monotonic, but showed a linear pattern.

**Figure 4:**
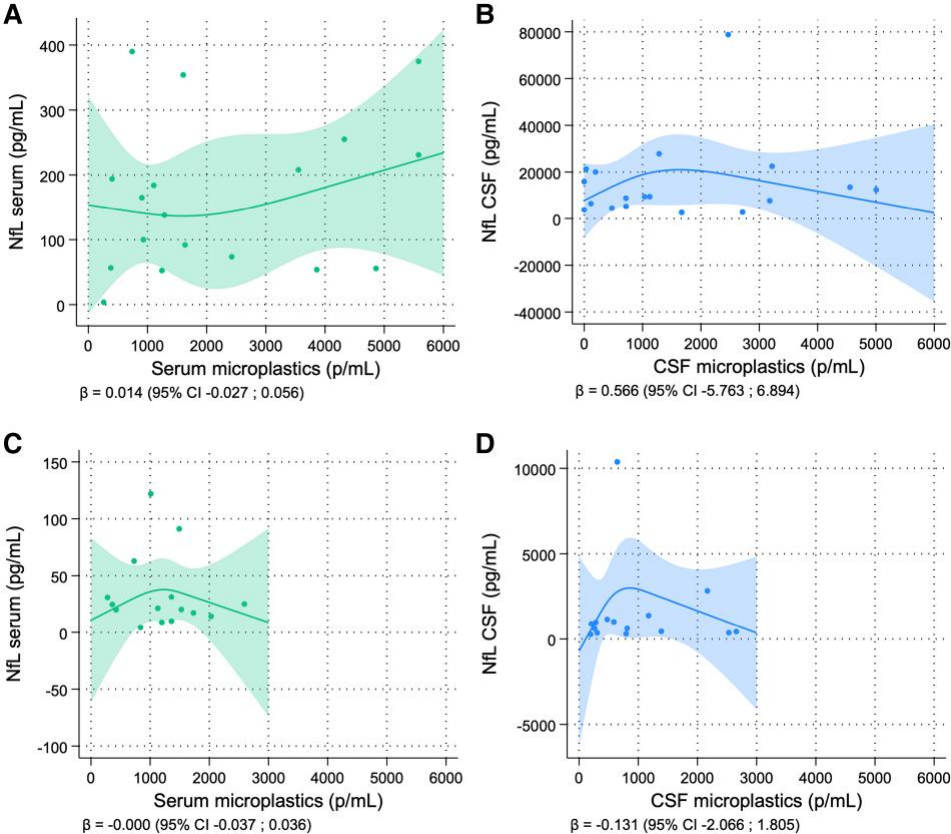
**Association of microplastic concentrations with NfL levels.** Serum and CSF microplastic concentrations and neurofilament (NfL) concentration in ALS patients (**A** and **B**, *N* = 24) and controls (**C** and **D**, *N* = 20). The solid lines indicate the multivariable analysis; the shaded areas represent upper and lower CI limits. Dots represent individual observations.

**Figure 5:**
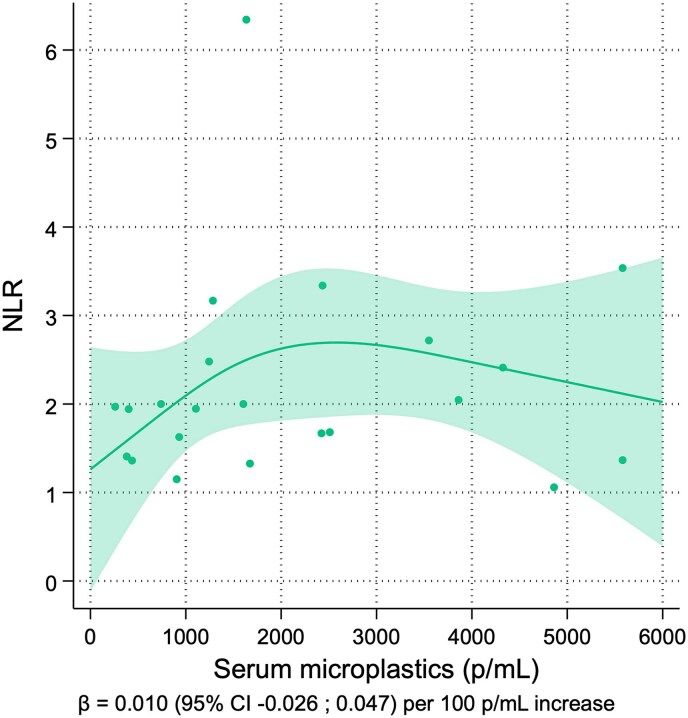
**Association of microplastic concentration with NLR.** Serum microplastic concentrations and serum NLR among ALS patients (*N* = 24). The solid line indicates the multivariable analysis; the shaded area represents upper and lower CI limits. Dots represent individual observations.

We also found a non-linear positive association between microplastic concentrations and NfL in serum of cases at the highest levels of exposure, while in the lowest exposure range, the relation was slightly inverse (Fig. 4). Conversely, the association between serum microplastics and NfL in controls, and between CSF microplastics and NfL both in cases and controls, showed an inverted U-shaped pattern (Fig. 4). NfL concentrations were much higher in cases compared with controls both in serum and CSF. No relation emerged for serum NLR depending on serum microplastic concentrations in cases (Fig. 5).

**Figure 6:**
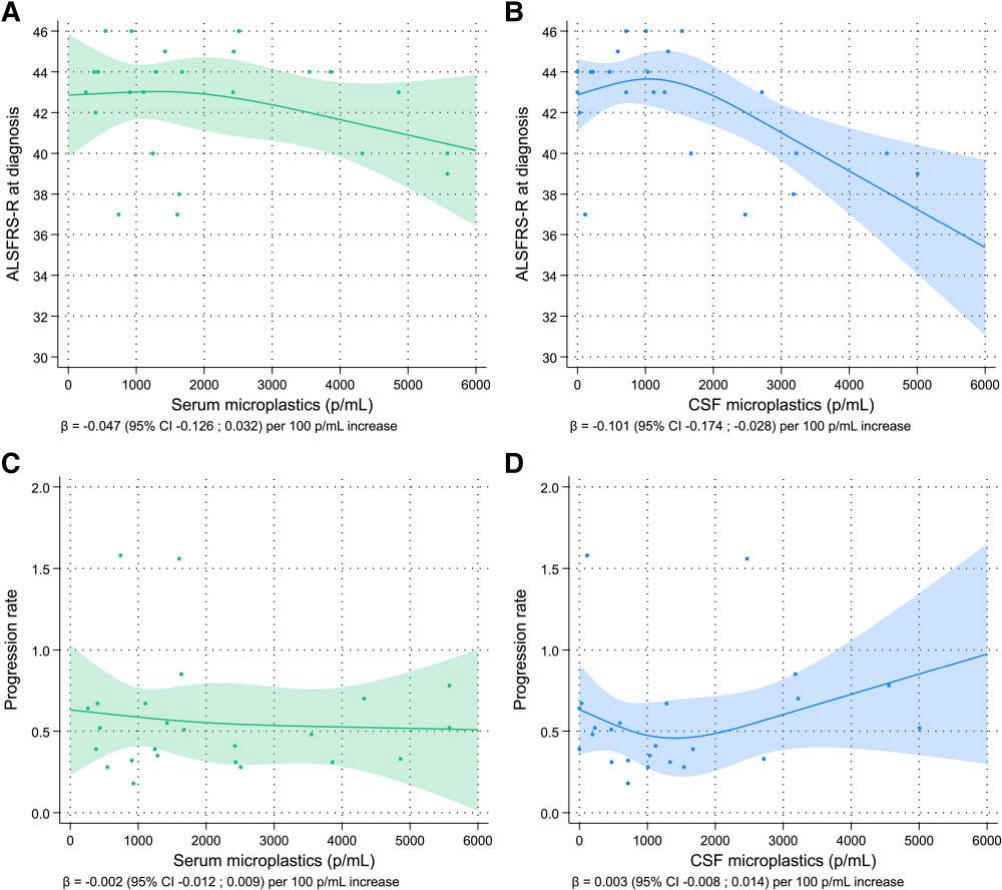
**Association of microplastic concentrations with ALSFRS-R and progression rate.** Serum and CSF microplastic concentrations in relation to ALS ALSFRS-R (**A** and **B**) and disease progression rate (**C** and **D**) among ALS patients (*N* = 24). The solid lines indicate the multivariable analysis; the shaded areas represent upper and lower CI limits. Dots represent individual observations.

Concerning disease progression, we did not identify consistent associations between microplastic concentrations above the median and tracheostomy-free survival during up to 85 months of follow-up (HR close to 1.00). We also observed no clear association between serum microplastic concentrations and progression rate, while cases with slightly faster progression rates had higher concentrations of CSF microplastics (Fig. 6). Serum microplastic concentrations displayed a weak inverse relation with ALSFRS-R scores at diagnosis, while CSF microplastic concentrations were associated with a steeper decrease in ALSFRS-R scores, indicating worse functional status at higher concentrations (Fig. 6).

## Discussion

To our knowledge, the present study is the first to report higher nano- and microplastic concentrations in the CSF and serum of patients newly diagnosed with amyotrophic lateral sclerosis in a human population. It is also among the very few *in vivo* reports of microplastic concentrations within the CNS. The association between microplastic biomarker concentrations and odds of amyotrophic lateral sclerosis was not linear, but it started only above a certain amount of exposure, regardless of whether this was assessed in serum or the brain.

Our analytical approach focused on microplastic particles sized from 0.1 to 10 µm, i.e. including part of nanoplastics (defined as included in the 0.001–1 µm^1,2,4^) and ‘small microplastics’, but not the entire broader particle category generally included in the ‘microplastics’ category, i.e. having a size <5 mm^2,3^ or from 1 to 1000 µm.^4^ Given the non-polar solvent used in the analytical phase, we could assume a substantial reduction of electrostatic interactions between polymer surfaces, thus preventing the formation of electrostatic bridges or differential repulsion that would otherwise promote particle aggregation processes. As a result, the particles remain predominantly isolated and well dispersed within the solvent matrix, as confirmed by microscopic observations showing single, non-associated morphologies, thus avoiding aggregation in such a non-polar environment.^36,37^ Plastic particles as small as 0.1–10 µm are not commonly measured, being below the detection limit most often used in analytical equipment.^38^ Consideration of very small-sized particles (i.e. nanoplastics) is important because ‘toxicity and the relative ease with which microplastics cross biological barriers are expected to increase with decreasing size,’^38^ and there is clear evidence that small particles can be more neurotoxic than large particles.^13^ Thus, our study addresses a size component of microplastic exposure that may have greater mechanistic relevance to neurodegeneration, crossing biological barriers and exerting cellular toxicity in the CNS, as also supported by toxicological evidence.^10,13,39-42^ There is also evidence that micro- and nanoplastics may induce endothelial dysfunction, oxidative stress and inflammatory signalling, or lead to formation of protein corona around their surface, in such a way affecting inflammatory, metabolic, barrier-related and other systemic pathways involved in onset of amyotrophic lateral sclerosis.^8,16,21,43-49^ The indication of a threshold effect in serum suggests that a critical systemic burden of microplastics needs to be reached to allow their crossing of the blood–brain barrier and the other brain barriers, thus raising risk of amyotrophic lateral sclerosis,^50^ while CSF microplastics, directly reflecting CNS exposure, result in a more linear association with disease occurrence. A finding of interest in our study was the lack of association between serum and CSF particle size, suggesting that the microplastics of the largest size do not enter the CNS, likely being blocked by the blood–brain barrier, not only in controls but also in patients with amyotrophic lateral sclerosis, notwithstanding the ongoing neurodegenerative process in the latter, primarily affecting motor neurons.

We found that microplastic levels were associated with slightly increased serum NfL concentrations among cases of amyotrophic lateral sclerosis only above a certain amount of exposure, possibly suggesting a threshold effect, and that a critical systemic burden may be required before measurable neuroaxonal injury becomes evident. NfL is a structural component of the axonal cytoskeleton that is released into biological fluids following axonal injury, representing a marker of neuroaxonal damage rather than a disease-specific marker of neurodegeneration. However, the slight NfL decrease in the lowest range of exposure and the inverted U-shaped pattern of association characterizing such relation in CSF among cases and in both serum and CSF among controls suggest caution in inferring any causal relation between microplastics and this biomarker, supporting if any no substantial effects of microplastics in contributing to a specific neurodegenerative process characterizing amyotrophic lateral sclerosis, the axonal degeneration of motor neurons down the lateral corticospinal tract.

There are several ‘caveats’ about the internal validity of results pertaining to microplastic exposure and amyotrophic lateral sclerosis in this study. First, given the case-control design of the study, we cannot determine whether higher CNS microplastic concentrations preceded or were a consequence of the disease (or its diagnostic procedures or treatments), thus hampering the assessment of the causal nature of this association.^19^ Disease status could substantially alter both blood–brain permeability and lifestyle of the individual and therefore theoretically favour greater flow of toxic chemicals and microplastics themselves into the CNS.^8,47^ In our study, however, cases were recruited at an early stage of the disease, i.e. during the diagnostic process, thereby reducing the potential for reverse causation. In addition, we also found an increased microplastic content in serum, a finding contradicting the possibility that our observations were due to an effect of amyotrophic lateral sclerosis, as conceivable for the higher CSF microplastic content.

Second, we cannot rule out residual or unmeasured confounding. As with all observational study designs, unmeasured confounding is possible, given that we did not measure or adjust for the many environmental chemicals and other factors potentially involved in disease aetiology. Microplastics, and more specifically nanoplastics, could represent just one component of a complex mixture of environmental exposures interacting with other neurotoxic chemicals or simply reflecting or favouring their presence in the CNS, therefore reflecting broader exposure patterns rather than a specific aetiologic factor. Such findings may however explain an increase in cases as an additional environmental risk factor. The long-standing historical presence of amyotrophic lateral sclerosis, preceding widespread plastic production, further argues against a simple causal model. In addition, we could not adjust the entire analysis for BMI due to the lack of comprehensive data in controls, though the available data indicated little difference with cases. BMI has been suggested to be positively associated with microplastic exposure^51,52^; however, in our results for cases and the few controls with such information, we found an indication of an inverse association.

Due to insufficient availability of the serum and CSF samples, we could not characterize the specific plastic polymers in CSF or serum, a major limitation. Recently developed analytical methods, such as pyrolysis–gas chromatography–mass spectrometry, could be of considerable utility to characterize polymers, though their use in lipid-rich matrices, such as serum, may generate interferences and analytical background noise, thus calling for the use of refined chemical digestion and recovery protocols.^53,54^ The specific neurotoxicity of the different microplastic polymers, and their translocation into the CNS, might considerably differ, also taking into account the extent of the blood–brain barrier impairment.^16^ Still, given the limited sample size, we also could not perform meaningful subgroup analysis according to the amyotrophic lateral sclerosis type of disease onset, though microplastic levels in the four patients for bulbar onset were rather similar to those characterizing patients with spinal onset. A larger number of participants would therefore be needed to allow for this (and other) stratified analyses.

In controls, we also could not entirely rule out the presence of subtle, preclinical neurodegenerative processes, also given the unavailability of CSF biomarkers apart from NfL. However, all controls underwent comprehensive clinical and diagnostic evaluation, and there was no indication of neurodegenerative disease.

Genetic heterogeneity may further contribute to inter-individual variability in the effects of environmental factors. In our cohort, however, only two cases carried known ALS-associated mutations (*SOD1* and *C9orf72*), precluding meaningful subgroup analyses but suggesting that genetic susceptibility is unlikely to have affected study findings. In addition, although the *SOD1*-mutated case exhibited relatively high microplastic levels, this isolated observation should be interpreted with caution and does not seem to allow inference on gene–environment interactions.

In two cases, we could not detect microplastics in CSF, despite measurable concentrations in serum, and no evidence of technical artefacts. This finding suggests that systemic exposure does not necessarily translate into CNS accumulation, reflecting inter-individual in blood–brain barrier permeability or in the clearance of microplastics within the CNS.^41,55,56^ These observations indicate possible heterogeneity in the relation between systemic and central microplastic burden and are consistent with threshold-like patterns, suggesting that CNS accumulation may occur only above a certain overall exposure level. In addition, the lack of microplastic detection in two cases, alongside the small size of our study population, further suggests caution in claiming a causal role of these contaminants in aetiology of amyotrophic lateral sclerosis.

Microplastics/nanoplastics could theoretically induce toxic mechanisms that either led to or triggered onset of amyotrophic lateral sclerosis through pathophysiological mechanisms, such as inflammation, oxidative stress, neurotransmitter dysfunction, neuronal damage and TDP-43 mislocalization and aggregation.^14,16,18,21,57,58^ Microplastics could also directly damage the blood–brain barrier, thereby increasing its permeability to chemicals and other substances.^18^ Microplastics can carry metals and metalloids adsorbed to their surfaces,^59^ thus potentially increasing CNS exposure to neurotoxic elements, such as lead, copper and selenium, or other chemicals potentially involved in aetiology of amyotrophic lateral sclerosis.^60-65^ Overall, microplastics might therefore act as vectors or amplifiers of environmental risk factors for amyotrophic lateral sclerosis while not having a direct toxic impact on motor neurons. Finally, the effect estimates we computed were imprecise, given the limited number of participants.

Study strengths include the *in vivo* measurement of microplastics in blood and CSF. We observed a strong and almost linear correlation between these matrices, suggesting systemic–central coupling of exposure to these exogenous chemicals and the validity of both biomarkers. Our finding differs from that yielded by the only previous study investigating paired biomarkers, which was unable to find an association in control subjects (differently from patients with neuroimmune and neuroinfectious disease), although that study was based on only six healthy participants and five types of microplastics.^11^ Finally, study participants were sampled from the same source population (i.e. both cases and controls from the two provinces of Modena and Reggio Emilia), thus reducing the potential for selection bias, but also suggesting that the external validity of our findings should be confirmed by studies carried out in other geographical settings. In fact, there is a large variability and disparity in the dietary intake and body burden of microplastics across the world,^66^ indicating both geographical priorities for efforts aiming at microplastic pollution mitigation and the opportunity to investigate the possible association of these contaminants with human diseases, including amyotrophic lateral sclerosis. Further research is also warranted about the actual relation of microplastic exposure with amyotrophic lateral sclerosis and more generally human neurodegenerative disease affecting both the motor and the cognitive–behavioural domain. Such studies should address key issues, such as the critical thresholds of exposure and the size and types of plastic polymers involved and eventually the potential for confounding that may affect such association.

## Data Availability

Data availability and sharing is not applicable due to confidentiality issues to not compromise the privacy of research participants and as per Ethics Committee policy. The STATA-19 code used for data analysis is available at https://git.unimore.it/creagen1/microplastics.
